# Utilization of home- and community-based services among rural family caregivers of persons with dementia: the role of the area deprivation index

**DOI:** 10.3389/fpubh.2025.1688161

**Published:** 2025-10-14

**Authors:** Jasmine Santoyo-Olsson, Kenneth E. Covinsky, Jing Cheng, Dolores Gallagher Thompson, Veronica Yank

**Affiliations:** ^1^Division of Geriatrics, University of California San Francisco, San Francisco, CA, United States; ^2^Division of Oral Epidemiology and Dental Public Health, University of California San Francisco, San Francisco, CA, United States; ^3^Department of Psychiatry and Behavioral Sciences, Stanford University, Palo Alto, CA, United States; ^4^Division of General Internal Medicine, University of California San Francisco, San Francisco, CA, United States

**Keywords:** social network, socially connected, activities of daily living, area-level factors, neighborhood atlas

## Abstract

**Background:**

Dementia significantly impacts rural communities in the U.S., presenting unique challenges for family caregivers due to limited access to essential support services. This study examines the influence of area-level factors on the utilization of home- and community-based services among rural caregivers.

**Methods:**

Using Andersen’s extended behavioral model of health services utilization, baseline data (*n* = 361) from family caregivers participating in a national randomized trial evaluating the *Building Better Caregivers* workshop were analyzed. Participants completed an online survey assessing home- and community-based support services and caregiving aspects. Area-level factors were measured using the Area Deprivation Index (ADI) and U.S. Census region, linked to respondents ZIP+4 code and state. Multivariable logistic regression analyses assessed the relationship between area-level factors and support service utilization.

**Results:**

About 65% of caregivers used at least one support service, with 52% utilizing home-based services and 52% community-based services. Caregivers in the most deprived rural areas were significantly less likely to use any support services (OR = 0.45; 95% CI [0.23, 0.89]) and community-based services (OR = 0.26; 95% CI [0.26, 0.86]) compared to those in the least deprived rural areas. No significant associations were found between Census region and any type of support service utilization.

**Conclusion:**

Service utilization differences within rural areas highlight the need for nuanced, area-specific interventions to enhance support service accessibility for caregivers in deprived rural areas, improving caregiving outcomes. Future research should further investigate area-level variations and their interactions with individual factors to better understand barriers faced by rural caregivers.

**Trial registration:**

https://clinicaltrials.gov/ct2/show/NCT04428112, identifier NCT04428112.

## 1 Introduction

Dementia affects a growing number of individuals in the United States (U.S.), posing significant challenges for family caregivers who are essential in managing the complex needs of people with dementia (PwD). Home- and community-based support services, such as respite care, home health aides, and specialized medical care, have been shown to improve the quality of life for both caregivers and PwD (1, 2). These services, defined as person-centered care delivered in the home and community to individuals with functional limitations who require assistance with daily activities (3). Despite the benefits of these services, many caregivers, particularly those in rural areas, report low levels of utilization (4).

Rural caregivers face unique challenges compared to urban counterparts, including fewer healthcare resources, reduced access to services, and diminished infrastructure to support caregiving (4). Area-level characteristics such as geographic isolation, transportation barriers, and socioeconomic conditions significantly shape access and use in rural settings (5). Understanding these challenges requires a comprehensive approach to identify specific individual- and area-level factors associated with support service utilization in varied rural settings.

Rural areas vary widely in economic conditions, health and social service infrastructures, natural amenities, and capacity to address economic and environmental stressors (6, 7). While some areas struggle with persistent poverty, high unemployment, out-migration, and poor health, others experience transformative demographic and economic changes driven by new industries and innovation (8). The Area Deprivation Index (ADI), a composite measure of socioeconomic deprivation (9), provides a granular lens for examining differences within rural areas. Capturing area-level factors such as poverty, education, housing, and employment (9), the ADI offers an alternative to broad rurality measures like the Urban Influence Code and Rural Urban Continuum Code, making it particularly relevant for understanding rural variability and its impact on support service utilization.

Existing research has examined support service utilization in relation to area-level factors, including U.S. Census region (4), Urban Influence Code (10), Rural Urban Continuum Code (11), residence in a specific rural area (e.g., Appalachia) (12–15), and county-level socioeconomic status (16). However, these broad measures often lack the granularity required to capture the nuanced intersection of socioeconomic deprivation and service utilization. The ADI allows for a more detailed understanding of how rural area-level factors impact support service utilization, filing an important gap in existing literature. While the ADI has been applied in studies investigating home-and community-based services use among veterans (17) and other types of services (18, 19), its application in the context of caregiver support services in rural areas remains novel. By leveraging the ADI at the ZIP+4 code level alongside U.S. Census regions, this study aims to offer a better understanding of rural variability and its influence on support service utilization.

Regional disparities in support service utilization further complicates this picture (4). The U.S. Census Bureau defines four regions: Northeast, Midwest, South, and West. Each region includes states with diverse demographic, economic, and healthcare infrastructure characteristics, which influence service availability and utilization. While regional comparisons provide helpful insights, they fail to account for rural-specific variability within regions. The ADI enables a more detailed analysis of area-level socioeconomic status, offering a multifaceted approach to understanding rural variability and its influence on support service utilization. Understanding these dynamics can inform targeted resource allocation and evidence-based policy development to improve caregiver support services for rural settings across the U.S.

This study is guided by Andersen’s extended behavioral model of health services utilization (20), which accounts for both individual- and area-level factors (what Andersen refers to as community-level factors in the model) influencing service use. Predisposing factors, such as demographic characteristics, influence individuals’ propensity to utilize services; need factors relate to health issues requiring care; and enabling factors, such as income, facilitate service use (21). Andersen’s extended model emphasizes that individual-level factors operate within broader area-level contexts, making it well-suited for analyzing the role of area-level deprivation and Census region in shaping service utilization (20).

Using data from a national randomized controlled trial (RCT) of rural caregivers of PwD, this study investigates the relationship between area-level socioeconomic status, geographic region, and the use of home- and community-based support services among caregivers of PwD residing in U.S. rural areas. We hypothesize that caregivers residing in most deprived rural areas (defined by higher ADI scores) or intermediate deprived rural areas will report lower support service utilization compared to those residing in the least deprived rural areas. We also hypothesize that rural caregivers residing in non-West U.S. Census regions (Midwest, Northeast, or South) will report lower utilization than those residing in the Western region (West), based on evidence suggesting higher service utilization in the West compared to other regions (4).

## 2 Methods

### 2.1 Study design and participants

This study is a cross-sectional secondary data analysis of baseline data from a national RCT evaluating an online skills-building workshop, *Building Better Caregivers*, for rural caregivers of PwD (*n* = 409). The parent RCT procedures and intervention are described elsewhere (22). Briefly, participants were recruited in collaboration with rural-serving community organizations. Inclusion criteria consisted of: adult self-identifying as living in a U.S. rural, farming, or small-town area; providing at least 10 caregiving hours per week to a family member or friend with dementia; reporting a stress level of 4+ on a 10-point scale (23); having internet access; and having English proficiency. Participants completed a baseline online survey that included assessments of home- and community-based support services and caregiving aspects. The study protocol was approved by the University of California, San Francisco Institutional Review Board.

This secondary analysis excluded caregivers who were non-relatives (*n* = 48). We focus on family caregivers (*n* = 361) because they may have access to family-based resources that are not available to non-relatives, such as shared housing or financial resources, that may influence support service utilization.

### 2.2 Measures

#### 2.2.1 Dependent measures

The dependent variables are self-reported current use of home- and community-based support services by caregivers or the PwD they assist. Home-based services include: homemaker assistance, non-medical personal care, home healthcare (e.g., nursing, hospice), and respite care (e.g., overnight respite). Community-based services include: meal delivery, transportation, adult day care, and use of informational services from a case manager/social worker or legal or financial services representative. Respondents answered “yes” or “no” to each service. For each service, “yes” responses were counted. If the count was 1 or greater, then the service was classified as 1 indicating use; otherwise, they were classified as 0. To determine use of any support service, respondents who answered “yes” to any type of service were classified as 1 indicating use; otherwise, they were classified as 0.

#### 2.2.2 Independent area-level measures

Independent variables were area-level factors: socioeconomic deprivation status and geographic Census region. Socioeconomic deprivation status was assessed using the Area Deprivation Index (ADI), a validated composite ranked index (9, 24) based on 17 indicators from the American Community Survey: educational distribution (percentage of population with less than 9 years versus 12 or more years of education), median family income, income disparity, occupational composition, unemployment rate, family poverty rate, percentage of population with income below 150% of the federal poverty level, single-parent household rate, home ownership rate, median home value, median rent, median monthly mortgage, household crowding, and percentages of households without access to plumbing, telephone, or motor vehicle (9). Respondent mailing address Zip+4 code was linked to the 2020 ADI ranking retrospectively. ADI rankings range from 1 to 10, with higher values indicating the most deprived areas (9). Respondents were classified into three groups: those living in the most deprived areas (ADI ≥ 7), those in intermediate deprived areas (4 ≤ ADI ≤ 6), and those in the least deprived areas (1 ≤ ADI ≤ 3). Geographic region was determined by caregiver state of residence and categorized according to U.S. Census regions: Midwest, Northeast, South, and West.

#### 2.2.3 Individual-level measures

Models adjusted for individual-level predisposing, need, and enabling factors.

*Predisposing factor* measures included caregiver age in years categorized as younger (18–64 years) or older (65 + years), gender (woman, man, refused), caregiver race or ethnicity (White, Black/African American, Latino/Hispanic, Native American/American Indian/Alaska Native, Asian/Asian American, Native Hawaiian/Other Pacific Islander, mixed race, other race) and education (high school or less, technical school or associate’s degree/some college, college graduate or higher), relationship to the PwD (parent, spouse), and co-residence with their PwD (co-reside, do not co-reside). Respondents also reported the age and gender of the PwD.

*Need factor* measures were the everyday cognition level of the PwD, PwD functional limitations, and care burden. *Everyday cognition* was assessed using the 12-item Everyday Cognition (ECog-12) measure (25). The ECog-12 score was created by averaging items. Scores ranged from 1 to 4. Higher scores indicate more cognitive impairment.

*Functional limitations of PwD* was measured using a checklist of activities of daily living (ADLs). The 6-item ADL checklist included needing help getting out of bed, getting across a room, dressing, toileting, bathing, and eating. Responses were summed, with scores ranging from 0 to 6. ADL scores were categorized as meeting nursing home placement criteria (score ≥ 3) and not meeting criteria (score < 3) (26).

*Caregiver burden* was assessed with the 12-item Zarit Burden Inventory short form (27). Responses were summed, with scores ranging from 0 to 48. Scores were categorized as high burden (score > 20) or none-to-moderate burden (score ≤ 20).

The *enabling factor* measure was caregiver *social network,* assessed using the 6-item Lubben Social Network Scale (28). A total score was the sum of items, with scores ranging from 0 to 30. Scores were categorized as socially connected (score ≥ 13) or not socially connected (score < 12) (28).

### 2.3 Statistical analysis

Descriptive statistics of measures were obtained to summarize the data. Categorical variables are expressed as number of subjects and percentage. Continuous variables are presented as mean and standard deviation (SD). Analysis of variance (ANOVA), t test, and chi-squared test were, respectively, used to assess whether continuous and categorical variables differed by area deprivation levels and Census regions separately (tables not shown). Correlations between the different types of support services (any, home- and community-based), area-level factors, and individual-level factors (table not shown) were examined for multicollinearity and deemed multicollinear if the Variance Inflation Factor (VIF) exceeded 5. No multicollinearity was detected. To assess associations between area-level factors and support service utilization, we first conducted bivariate analysis for the area-level factors (e.g., area deprivation levels, Census regions) with the utilization of each type of support service using chi-squared tests and univariate logistic regression. Then, we conducted multivariable logistic regression analyses on the associations between area-level factors and support service utilization while controlling for individual level variables (predisposing, need, and enabling factors). Predisposing factors, caregiver race or ethnicity and education, were excluded from the analysis due to limited variability. Unadjusted and adjusted odds ratio (OR), 95% confidence interval (CI), and *p*-values were calculated. Statistical significance was defined at *p* < 0.05. Analyses were conducted using SAS 9.4.

## 3 Results

As shown in Table 1, among 361 family caregivers, the mean age was 63.4 (SD = 10.4). The majority were women (83%) and self-identified as White (87%). Over half (57%) had completed a college degree or higher. Fifty percent of caregivers provided care to a spouse, while the remaining 50 % cared for a parent. The majority (80%) co-resided with their PwD. A little less than two-thirds (64%) of caregivers reported being socially connected. Among PwD, the average age was 79.1 (SD = 9.5) and 52% were women. The average level of cognition was 3.3 (SD = 0.7) indicating moderate or greater impairment, and 37% needed assistance with 3 or more ADLs. Almost two-thirds (64%) of caregivers reported high care burden.

**Table 1:** Descriptive characteristics of family caregivers and persons with dementia and key study variables (*N* = 361).

Mean (SD; range)	Total
Caregiver	
Age	63.4 (10.4; 30–86)
Gender, *n* (%)	
Women	299 (83%)
Men	58 (16%)
Prefer not to answer	4 (1%)
Race or ethnicity, *n* (%)	
White	315 (87%)
Black or African American	13 (4%)
Hispanic or Latino	13 (4%)
American Indian/Alaska Native	6 (2%)
Other^a^	14 (3%)
Education, *n* (%)	
Less than college graduate	156 (43%)
College graduate with bachelor’s degree or higher	205 (57%)
Relationship to person with dementia, *n* (%)	
Spouse	180 (50%)
Parent	181 (50%)
Co-resides with person with dementia, *n* (%)	
Yes	288 (80%)
No	73 (20%)
Care burden, *n* (%)	
None to moderate	129 (36%)
High	232 (64%)
Socially connected, *n* (%)	
Yes	231 (64%)
No	130 (36%)
Area deprivation level, *n* (%)	
Least deprived rural areas	75 (21%)
Intermediate deprived rural areas	102 (28%)
Most deprived rural areas	184 (51%)
U.S. Census region, *n* (%)	
West	124 (34%)
South	88 (24%)
Midwest	80 (22%)
Northeast	69 (19%)
Person with Dementia	
Age	79.1 (9.5; 52–100)
Gender, *n* (%)	
Women	188 (52%)
Men	173 (48%)
Level of cognitive impairment^b^	3.3 (0.7; 1.2–4.0)
Needs assistance with ≥3 activities of daily living, *n* (%)	
Yes	133 (37%)
No	228 (63%)
Support services utilized	
Any service, *n* (%)	234 (65%)
Home-based services^c^, *n* (%)	189 (52%)
Community-based services^d^, *n* (%)	187 (52%)

SD refers to standard deviation.

a Other includes Asian/Asian American, Native Hawaiian/Other Pacific Islander, mixed race, and other race.

b Cognitive impairment, possible range 1–4, higher = worse.

c Home-based services include: homemaker services, non-medical personal care, respite care, and home health care.

d Community-based services include: meal delivery, transportation, adult day care, and informational services from a case manager/social worker or legal or financial services representative.

Over half (51%) of caregivers lived in the most deprived rural areas, 28% lived in intermediate deprived areas, and 21% lived in the least deprived areas. Caregivers were represented across all four Census regions: 34% West, 24% South, 22% Midwest, and 19% Northeast.

Approximately two-thirds (65%) of caregivers used at least one service (Table 1), and an equal percentage of caregivers utilized home-based services (52%) and community-based services (52%).

### 3.1 Bivariate results

**Figure 1:**
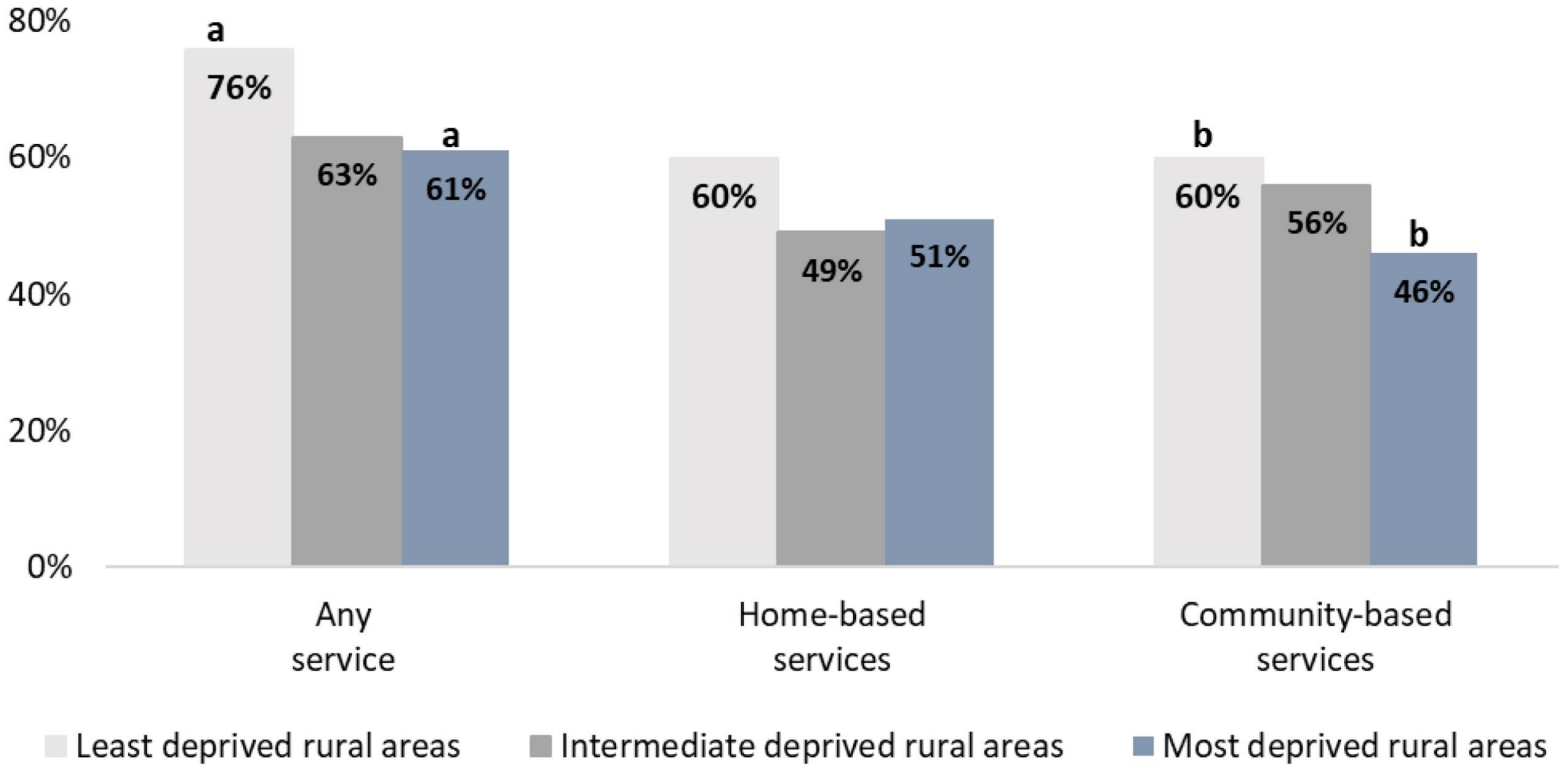
Support services utilized among family caregivers and persons with dementia by area deprivation index (*N* = 361). ^a,b^There is a statistically significant difference between the least and most disadvantaged areas for these services, with a *p* < 0.05. Home-based services include: homemaker services, non-medical personal care, respite care, and home health care. Community-based services include: meal delivery, transportation, adult day care, and informational services from a case manager/social worker or legal or financial services representative.

In bivariate analysis, we examined any support service use based on area deprivation level and Census region. As shown in Figure 1, caregivers in the least deprived rural areas reported the highest use of any service (76%), followed by those in intermediate deprived areas (63%) and those in the most deprived areas (61%). A statistically significant difference was observed between the least and most deprived rural areas (76% vs. 61%, *p* = 0.026). Caregivers residing in the most deprived areas had significantly lower odds of using any support service (OR = 0.48, *p* = 0.0201) compared to those residing in the least deprived areas.

For home-based services, caregivers in the least deprived rural areas reported the highest usage (60%), compared to similar rates in the intermediate deprived (49%) and most deprived (51%) areas. However, no statistically significant differences in the use of home-based services were found between the three deprivation groups (see Figure 1).

For community-based services, caregivers in the least deprived rural areas had the highest usage (60%), followed by those in areas with intermediate deprivation (56%) and those in the most deprived areas (46%). A statistically significant difference in the use of community-based services was observed between the least and most deprived rural areas (60% vs. 46%, *p* = 0.0449, see Figure 1). Caregivers residing in the most deprived areas (OR = 0.53, *p* = 0.0270) had significantly less odds to use community-based support services compared to those residing in the least deprived areas.

**Figure 2:**
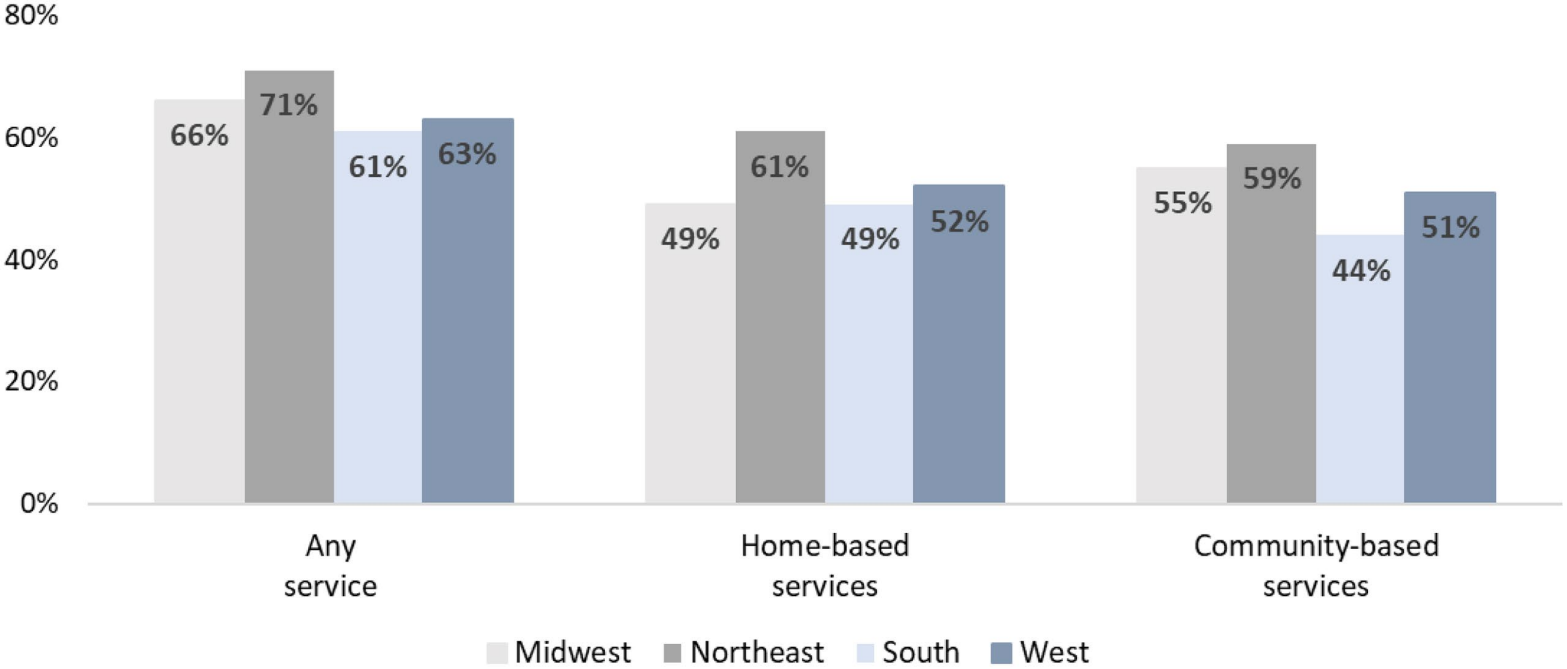
Support services utilized among family caregivers and persons with dementia by U.S. Census region (*N* = 361). Home-based services include: homemaker services, non-medical personal care, respite care, and home health care. Community-based services include: meal delivery, transportation, adult day care, and informational services from a case manager/social worker or legal or financial services representative.

As shown in Figure 2, caregivers in the Northeast region of the U.S. reported the highest use of any service (71%), home-based (61%), and community-based (59%) compared to all other regions. However, no statistically significant differences were found between the regions for utilization of each type of support services.

### 3.2 Multivariable results

Table 2 displays results from the adjusted multivariable logistic models on caregiver self-reported use of different types of support services.

**Table 2:** Unadjusted and adjusted odds ratio (OR) of using any, home-based, or community-based services among family caregivers of persons with dementia (*N* = 361).

Characteristic	Any service		Home-based services		Community-based services	
	Unadjusted OR (95% CI)	Adjusted OR (95% CI)	Unadjusted OR (95% CI)	Adjusted OR (95% CI)	Unadjusted OR (95% CI)	Adjusted OR (95% CI)
Intermediate deprived rural areas (ref. Least deprived rural areas)	0.54 (0.28, 1.05)	0.39 (0.19, 0.82)*	0.65 (0.35, 1.19)	0.52 (0.26, 1.03)	0.86 (0.47, 1.58)	0.68 (0.36, 1.32)
Most deprived rural areas (ref. Least deprived rural areas)	0.48 (0.26, 0.89)*	0.45 (0.23, 0.89)*	0.64 (0.37, 1.21)	0.63 (0.34, 1.19)	0.53 (0.30, 0.93)*	0.48 (0.26, 0.86)*
Northeast region (ref. West)	1.47 (0.78, 2.80)	1.04 (0.50, 2.14)	1.42 (0.78, 2.59)	1.00 (0.50, 1.99)	1.48 (0.81, 2.71)	1.15 (0.59, 2.24)
South region (ref. West)	0.88 (0.50, 1.56)	0.71 (0.37, 1.35)	0.84 (0.48, 1.46)	0.62 (0.33, 1.16)	0.70 (0.40, 1.22)	0.61 (0.33, 1.12)
Midwest region (ref. West)	1.06 (0.58, 1.94)	1.04 (0.50, 2.14)	0.82 (0.46, 1.46)	1.00 (0.50, 1.99)	1.05 (0.59, 1.86)	0.94 (0.50, 1.76)
≥ 65 years old (ref. 18–64 years old)	–	1.60 (0.87, 2.92)	–	1.11 (0.62, 1.97)	–	1.52 (0.86, 2.67)
Women (ref. Men)	–	0.46 (0.23, 0.93)*	–	0.36 (0.19, 0.71)**	–	0.57 (0.31, 1.06)
Parent (ref. Spouse)	–	2.67 (1.37, 5.18)**	–	3.04 (1.59, 5.80)***	–	2.05 (1.11, 3.80)*
Co–resides with person with dementia (ref. Does not co–reside)	–	0.47 (0.22, 0.99)*	–	0.78 (0.40, 1.52)	–	0.44 (0.23, 0.85)*
Cognitive impairment	–	1.38 (0.93, 2.02)	–	1.43 (0.97, 2.11)	–	1.20 (0.83, 1.74)
Person with dementia needs assistance with ≥3 ADL (ref. Needs assistance with < 3 ADLs)	–	3.09 (1.73, 5.53)***	–	3.81 (2.22, 6.56)****	–	1.74 (1.05, 2.90)*
High care burden (ref. None to moderate burden)	–	1.08 (0.65, 1.81)	–	0.91 (0.55, 1.52)	–	1.58 (0.98, 2.56)
Socially connected (ref. Not connected)	–	1.80 (1.08, 3.01)*	–	1.94 (1.17, 3.24)*	–	1.72 (1.06, 2.78)*

CI, confidence interval; ref, reference; ADLs, activities of daily living.

*p < 0.05; **p < 0.01; ***p < 0.001; ****p < 0.0001.

Home-based services include: homemaker services, non-medical personal care, respite care, and home health care.

Community-based services include: meal delivery, transportation, adult day care, and informational services from a case manager/social worker or legal or financial services representative.

#### 3.2.1 Utilization of any support service

In adjusted models, caregivers residing in intermediate deprived rural areas (OR = 0.39, *p* = 0.014) and those residing in the most deprived rural areas (OR = 0.45, *p* = 0.021) both had significantly lower odds of utilizing any support services compared to caregivers in the least deprived areas. The odds of utilizing any support service was not associated with any Census region. Among individual-level factors, the odds of using any support service were lower for women (OR = 0.46, *p* = 0.029) compared to men and those co-residing with their PwD (OR = 0.47, *p* = 0.049) compared to those not co-residing. Caregivers caring for a parent (OR = 2.67, *p* = 0.004) compared to those caring for a spouse had significantly higher odds of using any support service. Similarly, caregivers caring for a PwD requiring assistance with 3 or more ADLs (OR = 3.09, *p* = 0.0001) compared to those supporting a PwD requiring assistance with < 3 ADLs had significantly higher odds of using any support service. Caregivers socially connected (OR = 1.80, *p* = 0.025) compared to those not socially connected had significantly higher odds of using any support service.

#### 3.2.2 Utilization of home-based support services

In adjusted models, utilizing home-based support services was not associated with either area-level deprivation or any Census region. Among individual-level factors, the odds of using home-based support services were lower for women (OR = 0.36, *p* = 0.003) compared to men. Caregivers caring for a parent (OR = 3.04, *p* = 0.001) compared to those caring for a spouse had significantly higher odds of using home-based support services. Caregivers caring for a PwD requiring assistance with 3 or more ADLs (OR = 3.81, *p* < 0.0001) compared to those supporting a PwD requiring assistance with < 3 ADLs had significantly higher odds of using home-based support services. Caregivers socially connected (OR = 1.94, *p* = 0.011) compared to those not socially connected had significantly higher odds of using home-based support services.

#### 3.2.3 Utilization of community-based services

In adjusted models, caregivers residing in the most deprived rural areas (OR = 0.48, *p* = 0.015) had significantly lower odds of using community-based support service compared to those residing in the least deprived rural areas. The odds of utilizing community-based support services was not associated with any Census region. Among individual-level factors, the odds of using community-based support services was lower for caregivers co-residing with their PwD (OR = 0.44, *p* = 0.015) compared to those not co-residing. Caregivers caring for a parent (OR = 2.05, *p* = 0.022) compared to those caring for a spouse had significantly higher odds of using community-based support services. Caregivers caring for a PwD requiring assistance with 3 or more ADLs (OR = 1.74, *p* = 0.033) compared to those supporting a PwD requiring assistance with < 3 ADLs had significantly higher odds of using community-based support services. Caregivers socially connected (OR = 1.72, *p* = 0.027) compared to caregivers not socially connected had significantly higher odds of using community-based support services.

## 4 Discussion

Our study investigated the utilization of home- and community-based support services among rural caregivers of PwD, focusing on the influence of area-level factors: socioeconomic deprivation status and geographic Census region. Findings highlight significant variation in service utilization across rural areas. Caregivers residing in the most deprived rural areas were substantially less likely to use any support services and community-based services compared to those in the least deprived rural areas. These results align with our hypotheses, underscoring the crucial relationship between area-level deprivation status and successful utilization of services. While area-level deprivation did not predict the utilization of home-based support services, the observed effect size suggests a potentially meaningful relationship that warrants further investigation.

Contrary to initial hypotheses, our study found no significant association between geographic Census region and support service utilization, which contrasts with previous research (4). This suggests that socioeconomic status within rural areas is a more critical determinant of service utilization than regional Census location. Our use of ADI offered a detailed measure of area-level socioeconomic status, enabling a more nuanced understanding of its impact on service utilization. Additionally, focusing on granular-level data, such as ZIP+4 codes, rather than larger regions, provides a more accurate depiction of rural area-specific disparities. Our findings align with previous research on hospital utilization suggesting that utilizing smaller geographic levels enhances measurement precision and may result in targeted policies that more effectively address rural disparities (29). Future research should delve deeper into specific area-level variations and their interactions with socioeconomic factors to better understand the complex barriers faced by rural caregivers.

Our analysis further underscores the complexity within rural classifications, demonstrating that not all rural areas face the same level of deprivation. Rural areas vary significantly in terms of health and social service infrastructures, economic conditions, and natural amenities (6, 7). The current analysis suggests that caregivers in the most deprived rural areas face heightened barriers to accessing support services, likely due to persistent poverty, high unemployment, and limited healthcare resources (8). In contrast, caregivers in the least deprived rural areas have access to better infrastructure and more services. Examining other area-level factors such as local healthcare infrastructure and community resources could provide further insights into improving support service accessibility, especially for communities most in need.

At the individual-level, several factors were significantly associated with support service utilization. Women were less likely to use both any support services and home-based support services compared to men. This may reflect gender differences in caregiving roles and perceived need for external support (6, 30). Additionally, caregivers who co-resided with PwD were less likely to use services, which might be due to practical challenges (e.g., transportation issues, time constraints) in accessing services or a greater reliance on informal care within the household.

Caregivers providing care to parents, those caring for individuals needing assistance with three or more ADLs, and those who reported being socially connected were more likely to use support services. Prior studies have found that caregivers reporting having a social network, family support, or social support were more likely to utilize services early in the dementia trajectory (12, 31, 32). Our findings are similar in that the presence of social connectedness was associated with higher service utilization. However, other studies have noted that social networks diminish as dementia progresses, resulting in underutilization of support services (33, 34). Although our study did not assess the duration of dementia, more than half of PwD in our study exhibited moderate–severe cognitive impairment, indicating they were in the later stages of disease and may have needed assistance with three or more ADLs. These findings highlight the importance of social support networks and degree of cognitive impairment as drivers of service utilization. Interventions aimed at enhancing social connectivity and addressing caregiving burdens related to cognitive impairment may improve service use.

Our study did not measure factors such as falls, hospitalizations, or other clinical indicators that may prompt clinician orders for home-based services. These orders, often tied to insurance (e.g., Medicare) benefits, could promote access to supportive services, particularly in areas of deprivation. As such, our findings indicating that persons requiring assistance with three or more ADLs had greater odds of using any supportive service should be interpreted with caution. Future research should include clinical variables, along with insurance coverage criteria, to better understand their impact on service uptake.

This study underscores the complex interplay between individual-level and area-level factors in shaping the utilization of support services among rural caregivers. The findings have important implications for research and practice. Longitudinal studies are needed to understand how changes over time in ADI-level socioeconomic conditions influence support service utilization patterns. Additionally, qualitative research could provide deeper insights into caregiver social connectivity, burden, and perceptions of support services. These findings could inform more targeted interventions, such as content for online caregiver support programs or mobile app-based solutions. Tailored approaches that account for individual caregiver characteristics and needs may improve service utilization and enhance the quality of life for both caregivers and PwD.

To address disparities in service utilization, the ADI can be leveraged to identify caregivers in high-deprivation areas and help connect them with accessible resources. Promoting support services through local media (e.g., radio, newspapers) can increase visibility and engagement. Additionally, outreach initiatives, such as distributing informational flyers, posters, and newsletters through local businesses, community organizations, schools, and healthcare providers, can further enhance awareness of available services. Strengthening referral systems is a critical step in this effort. Healthcare systems and local aging services organizations can maintain up-to-date directories of local services, including home health aides, transportation assistance, meal delivery programs, and caregiver support groups, to provide timely and accurate referrals. ADI data can also be used to identify regions with high levels of deprivation and determine whether or not existing home- and community-based support services serve those areas, which would allow resources to be more effectively targeted to areas of greatest need.

This study included a geographically diverse sample of rural caregivers but has limitations to consider. The cross-sectional design of the study precludes the ability to infer causality between area-level factors and support service use. The parent RCT was not powered to detect possible differences among different service utilization factors. Caregivers who participated in the parent RCT may differ from those who did not enroll with respect to service utilization patterns, ability to complete an online survey, cultural differences, health status, or other unmeasured characteristics. Participants were not asked to report additional details about the services they received, e.g., agency providing service, service duration, reason for utilizing service (dementia versus other reason), or insurance eligibility. Self-reported utilization was not independently confirmed. This study did not assess the number or accessibility of services in caregivers’ geographic area. Future research could benefit from using in-person recruitment methods, expanding the range of variables influencing service utilization, conducting qualitative interviews to explore caregiver experiences with service utilization, and mapping service availability in rural communities to better capture the realities of caregiving in rural areas.

Our study provides valuable insights into the disparities in support service utilization among rural caregivers of PwD, highlighting the significant associations between area deprivation level and service use. Addressing these disparities through targeted research and area-specific interventions is crucial for improving the quality of life for both caregivers and PwD in rural areas. By focusing on variations in area deprivation within rural regions, future efforts can more effectively bridge the gap in service utilization and support the needs of caregivers in rural areas.

## Data Availability

The data analyzed in this study is subject to the following licenses/restrictions: The data that support the findings of this study are available from the corresponding author upon reasonable request. Requests to access these datasets should be directed to Jasmine Santoyo-Olsson, jasmine.santoyo-olsson@ucsf.edu.

## References

[1] Williams F Moghaddam N Ramsden S De Boos D. Interventions for reducing levels of burden amongst informal carers of persons with dementia in the community. A systematic review and meta-analysis of randomised controlled trials. Aging Ment Health. (2019) 23:1629–42. doi: 10.1080/13607863.2018.1515886, PMID: 30450915

[2] Trivedi R Ngo V Lee T Humber MB Risbud R Jacobs JC. Barriers to accessing home and community-based services among family caregivers of veterans. J Am Geriatr Soc. (2024) 72:3541–50. doi: 10.1111/jgs.19051, PMID: 39319417

[3] Centers for Medicare & Medicaid Services. Home- and Community-Based Services Baltimore, MD; (2025). Available online at: https://www.cms.gov/training-education/partner-outreach-resources/american-indian-alaska-native/ltss-ta-center/information/ltss-models/home-and-community-based-services (Accessed February 7, 2025)

[4] Feldman SJ Solway E Kirch M Malani P Singer D Roberts JS. Correlates of formal support service use among dementia caregivers. J Gerontol Soc Work. (2021) 64:135–50. doi: 10.1080/01634372.2020.1816589, PMID: 32921273 PMC9048125

[5] Russell D Miyawaki CE Reckrey JM Bouldin ED. Unmet needs and factors impacting home- and community-based service use among rural Appalachian caregivers of people with Alzheimer’s and dementia. J Appl Gerontol. (2025) 44:628–37. doi: 10.1177/07334648241280041, PMID: 39263814 PMC11896892

[6] Innes A Morgan D Kosteniuk J. Dementia care in rural and remote settings: a systematic review of informal/family caregiving. Maturitas. (2011) 68:34–46. doi: 10.1016/j.maturitas.2010.10.002 21093996

[7] Buckwalter KC Davis LL. Elder caregiving in rural communities In: Talley RC Chwalisz K Buckwalter KC, editors. Rural caregiving in the United States. New York: Springer (2011). 33–46.

[8] Kerlin M O’Farrell N Riley R Schaff R. Rural rising: Economic development strategies for America’s heartland. New York: McKinsey & Company (2022).

[9] Kind AJH Buckingham WR. Making neighborhood-disadvantage metrics accessible - the neighborhood atlas. N Engl J Med. (2018) 378:2456–8. doi: 10.1056/NEJMp1802313, PMID: 29949490 PMC6051533

[10] Beeber AS Thorpe JM Clipp EC. Community-based service use by elders with dementia and their caregivers: a latent class analysis. Nurs Res. (2008) 57:312–21. doi: 10.1097/01.NNR.0000313500.07475.eb, PMID: 18794715

[11] Yoshikawa A Bouldin ED Lopez-Anuarbe M Kindratt TB Sylvers DL Webster NJ. Use of caregiving support services among diverse dementia caregivers by geographic context. The Gerontologist. (2024) 64:64. doi: 10.1093/geront/gnad067, PMID: 37318017 PMC10825843

[12] Gibson A Holmes SD Fields NL Richardson VE. Providing care for persons with dementia in rural communities: informal caregivers’ perceptions of supports and services. J Gerontol Soc Work. (2019) 62:630–48. doi: 10.1080/01634372.2019.1636332, PMID: 31250733

[13] Roberto KA Savla J McCann BR Blieszner R Knight AL. Dementia family caregiving in rural Appalachia: a sociocultural model of care decisions and service use. J Gerontol B Psychol Sci Soc Sci. (2022) 77:1094–104. doi: 10.1093/geronb/gbab236, PMID: 34951643 PMC9159069

[14] Savla J Roberto KA Blieszner R Knight AL. Family caregivers in rural Appalachia caring for older relatives with dementia: predictors of service use. Innov Aging. (2022) 6:igab055. doi: 10.1093/geroni/igab055, PMID: 35146130 PMC8824523

[15] Vipperman A Savla J Roberto KA Burns D. Barriers to service use among dementia family caregivers in rural Appalachia: implications for reducing caregiver overload. Prev Sci. (2023) 24:950–60. doi: 10.1007/s11121-022-01479-w, PMID: 36543967 PMC9771774

[16] Nah S Savla J Roberto KA. Dementia Care in Rural Appalachia: multilevel analysis of individual- and county-level factors. Gerontologist. (2024) 64:64. doi: 10.1093/geront/gnae037, PMID: 38661552 PMC11192855

[17] Huan T Intrator O Kind AJ Hartronft S Kinosian B. Provision of home & community based services to veterans by race, rurality, and neighborhood deprivation index. J Aging Soc Policy. (2024) 37:1–18. doi: 10.1080/08959420.2024.2402110 39369339

[18] Morenz AM Wong ES Zhou L Chen CP Zerzan-Thul J Liao JM. Neighborhood socioeconomic disadvantage and acute care utilization in Washington state Medicaid: a retrospective cohort study. J Gen Intern Med. (2025) 40:595–602. doi: 10.1007/s11606-024-09114-w, PMID: 39394471 PMC11861471

[19] Wang F Zeng Y Liu L Onega T. Disparities in spatial accessibility of primary care in Louisiana: from physical to virtual accessibility. Front Public Health. (2023) 11:1154574. doi: 10.3389/fpubh.2023.1154574, PMID: 37143988 PMC10151773

[20] Andersen R Davidson P. Improving access to care in America: Individual and contextual indicators. Changing the US health care system: Key issues in health services policy and management (2007)

[21] Andersen RM. Revisiting the behavioral model and access to medical care: does it matter? J Health Soc Behav. (1995) 36:1–10. doi: 10.2307/2137284 7738325

[22] Santoyo-Olsson J Lorig K Romo EM Luzanilla M Ramirez GA Cheng J. Study protocol for a hybrid effectiveness-implementation trial of the building better caregivers online workshop for rural family/friend caregivers of people living with dementia. Contemp Clin Trials. (2022) 121:106903. doi: 10.1016/j.cct.2022.106903, PMID: 36057375 PMC10292579

[23] Lorig K Stewart A Ritter P González V Laurent D Lynch J. Outcome measures for health education and other health care interventions. Thousand Oaks, CA, US: Sage Publications, Inc (1996). 99 p.

[24] University of Wisconsin School of Medicine and Public Health. Area Deprivation Index v4.0 2024. Available online at: https://www.neighborhoodatlas.medicine.wisc.edu/.

[25] Tomaszewski Farias S Mungas D Harvey DJ Simmons A Reed BR Decarli C. The measurement of everyday cognition: development and validation of a short form of the everyday cognition scales. Alzheimers Dement. (2011) 7:593–601. doi: 10.1016/j.jalz.2011.02.007, PMID: 22055976 PMC3211103

[26] Deardorff WJ Jeon SY Barnes DE Boscardin WJ Langa KM Covinsky KE. Development and external validation of models to predict need for nursing home level of Care in Community-Dwelling Older Adults with Dementia. JAMA Intern Med. (2024) 184:81–91. doi: 10.1001/jamainternmed.2023.6548, PMID: 38048097 PMC10696518

[27] Bedard M Molloy DW Squire L Dubois S Lever JA O’Donnell M. The zarit burden interview: a new short version and screening version. Gerontologist. (2001) 41:652–7. doi: 10.1093/geront/41.5.652, PMID: 11574710

[28] Lubben J Blozik E Gillmann G Iliffe S von Renteln KW Beck JC. Performance of an abbreviated version of the Lubben social network scale among three European community-dwelling older adult populations. The Gerontologist. (2006) 46:503–13. doi: 10.1093/geront/46.4.503, PMID: 16921004

[29] Buckingham WR Ryan Powell W Keller SA Hansmann KJ Kind AJ. Bigger isn’t better: why small area geographies are best for actionable index development. Pap Appl Geogr. (2024) 10:89–95. doi: 10.1080/23754931.2024.2312192, PMID: 39171071 PMC11335328

[30] Bayly M Morgan D Froehlich Chow A Kosteniuk J Elliot V. Dementia-related education and support service availability, accessibility, and use in rural areas: barriers and solutions. Can J Aging. (2020) 39:545–85. doi: 10.1017/S0714980819000564, PMID: 31975685

[31] Ehrlich K Emami A Heikkila K. The relationship between geographical and social space and approaches to care among rural and urban caregivers caring for a family member with dementia: a qualitative study. Int J Qual Stud Health Well Being. (2017) 12:1275107. doi: 10.1080/17482631.2016.1275107, PMID: 28452593 PMC7011969

[32] Orpin P Stirling C Hetherington S Robinson A. Rural dementia carers: formal and informal sources of support. Ageing Soc. (2014) 34:185–208. doi: 10.1017/S0144686X12000827, PMID: 24453384 PMC3894066

[33] Ervin K Reid C. Service utilisation by carers of people with dementia in rural Victoria. Australas J Ageing. (2015) 34:E1–6. doi: 10.1111/ajag.12162, PMID: 25427659

[34] Forbes D Ward-Griffin C Kloseck M Mendelsohn M St-Amant O DeForge R. “Her world gets smaller and smaller with nothing to look forward to” dimensions of social inclusion and exclusion among rural dementia care networks. Online J Rural Nurs Health Care. (2011) 11:27–42. doi: 10.14574/ojrnhc.v11i2.18

